# New Oligomeric Dihydrochalcones in the Moss *Polytrichum commune*: Identification, Isolation, and Antioxidant Activity

**DOI:** 10.3390/metabo12100974

**Published:** 2022-10-14

**Authors:** Anna V. Faleva, Nikolay V. Ul’yanovskii, Danil I. Falev, Aleksandra A. Onuchina, Nikolay A. Budaev, Dmitry S. Kosyakov

**Affiliations:** Laboratory of Natural Compounds Chemistry and Bioanalytics, Core Facility Center “Arktika”, M.V. Lomonosov Northern (Arctic) Federal University, Northern Dvina Emb. 17, 163002 Arkhangelsk, Russia

**Keywords:** moss, bryophytes, polytrichum commune, secondary metabolites, polyphenols, dichydrochalcones, 3-hydroxyphloretin, antioxidant activity

## Abstract

One of the most widespread representatives of mosses in the temperate and boreal latitudes of the Northern Hemisphere is common haircap (*Polytrichum commune*), which is known as the largest moss in the world and widely used in traditional herbal medicine. Polyphenolic compounds constitute one of the most important groups of biologically active secondary metabolites of *P. commune*, however, the available information on their chemical composition is still incomplete and contradictory. In the present study, a group of dihydrochalcone polyphenolic derivatives that were not previously found in mosses was isolated from *P. commune* biomass using pressurized liquid extraction with aqueous acetone. The combination of two-dimensional NMR spectroscopy and high-performance liquid chromatography–high-resolution mass spectrometry allowed for identifying them as 3-hydroxyphloretin oligomers formed through a carbon–carbon bond between phloroglucinol and pyrocatechol moieties (“head-to-tail” coupling), with a polymerization degree of 2–5. The individual compounds isolated by preparative reverse-phase HPLC had a purity of 71 to 97% and demonstrated high radical scavenging activity (17.5–42.5% with respect to Trolox) determined by the photochemiluminescence method. Along with the low toxicity predicted by QSAR/QSTR algorithms, this makes 3-hydroxyphloretin oligomers a promising source for the production of biologically active food additives and pharmaceuticals.

## 1. Introduction

Mosses (Bryophytes) are the second largest group in the plant kingdom after flowering plants and include more than 20,000 species. They grow on all continents and make up about five percent of the total number of all plant species on Earth. Mosses are the most important component of the wetland ecosystems of the vast subarctic territories of Eurasia and North America and can be harvested in these regions on an industrial scale as a renewable source of various pharmaceutical substances, food additives, and cosmetics [1,2]. One of the most widespread representatives of mosses in the temperate and boreal latitudes of the Northern Hemisphere is *Polytrichum commune*, which belongs to the family *Polytrichum* and is also known as common haircap (Supplementary Material, Figure S1). Since the length of its stems reaches 50 cm in favorable conditions, *P. commune* is considered the largest moss in the world, and its biomass is readily available for harvesting. In folk medicine, *P. commune* is used as an antipyretic and antidote, to stop bleeding, and to treat cuts, pneumonia, and even pulmonary tuberculosis [3,4]. Moreover, in Chinese traditional herbal medicine, the dried whole plant is widely used to treat memory decline and other related diseases [5]. Despite this, *P. commune* has not yet been found to have application in modern pharmacology and other areas due to the complexity and poor knowledge of its chemical composition.

It is well known that the main groups of biologically active secondary metabolites in mosses are terpenes, steroids, cyanoglycosides, and various aromatic (phenolic) compounds [6,7,8]. The latter group is the most extensive and represented by flavonoids (especially in the dimeric form) and their glycosides, bibenzyl and bis(bibenzyl) derivatives, alkyl and aryl benzoates, coumarins, and monomeric aromatic acids (benzoic, protocatehuic, vanillic, ferulic, and caffeic) [5,6,8]. In mosses of the genus *Polytrichum*, unusual benzonaphthoxanthenones and cinnamoyl bibenzyls have been found, which exhibit anti-inflammatory properties and cytotoxic activity against a number of tumor cell lines [5,9]. Fu et al. [10], using column chromatographic separation of acetone extract and NMR analysis of the obtained fractions, identified specific bioactive metabolites from *P. commune*—a new benzonaphthoxanthenone ohioensin with pronounced anti-inflammatory properties [11] and two unusual flavanone–styryl hybrid compounds, which were called communins after the plant from which they were isolated. Along with these, known aromatic compounds such as 4-hydroxybenzoic and 3-methoxy-4-hydroxybenzoic (vanillic) acids, 5-hydroxy-7-methoxy-4H-chromen-4-one, and 5-hydroxy-6-methoxy-7-O-*β*-D-glucosyl coumarin were detected for the first time in *P. commune*. Due to the exceptionally high content of polyphenols in the methanolic extracts of this moss, they were characterized by their pronounced antioxidant properties, including exceptionally high superoxide radical scavenging activity [12].

One of the most important polyphenolic components of plant biomass is lignin. The question of its presence in mosses has been the subject of discussion for a long time and, perhaps, has not been unambiguously resolved so far. In early studies (1960s–1970s), the presence of lignin was established in some types of “giant” mosses, in which this polymer, according to the authors, possesses only a mechanical function, and all other bryophytes are characterized by its absence [13]. Later, the presence of lignin or other low molecular lignin compounds in mosses was refuted [14]. However, Karmanov et al. reported lignin isolation from *P. commune* that was similar to softwood lignins in many characteristics [15]. The most recent study by Rencoret et al. [16] points to the detection of weak signals belonging to the *p*-hydroxyphenyl structures typical for lignin in the NMR spectra of a lignin-like substance isolated from *P. commune*. However, it was noted that flavonoids or other polyphenolic compounds can also be their source.

Our preliminary screening studies of *P. commune* extracts by NMR spectroscopy have shown the presence of unknown phenolic compounds not described earlier. This, along with the incomplete and contradictory information available in the literature, prompted us to carry out this study aimed at the search, isolation, and identification of new phenolic compounds that potentially possess high biological activity and antioxidant properties. Considering the complexity of the object of study, to achieve the set goals, we have chosen a combination of complementary analytical techniques, including two-dimensional NMR spectroscopy, high-performance liquid chromatography-high-resolution mass spectrometry (HPLC-HRMS), as well as preparative HPLC.

## 2. Materials and Methods

### 2.1. Reagents and Materials

Hexane (chem. pure., grade 2) and acetone (chem. pure) were purchased from Cryochrom (St. Peterburg, Russia) and Khimmed (Moscow, Russia), respectively, and used for the plant biomass extraction. To remove impurities, the acetone was additionally purified by distillation. In HPLC separations, gradient grade acetonitrile (Cryochrom, St. Peterburg, Russia), ACS reagent grade formic acid (≥96%, Merck, Darmstadt, Germany), and ultrapure Type I water with a resistivity of 18.2 MΩ·cm obtained in the Milli-Q system (Millipore, Molsheim, France) were used for the mobile phase preparation. Deuterated dimethylsulfoxide (DMSO-*d*_6_) and methanol (CD_3_OD) with a purity of > 99.8%, purchased from Deutero GmbH (Kastellaun, Germany), were used in NMR analysis. HPLC grade methanol (Khimmed, Moscow, Russia) was used as a solvent in photochemiluminescence antioxidant activity determination and as a sample preparation in preparative HPLC separations.

### 2.2. Plant Material and Extraction

Samples of the moss *P. commune* (Supplementary Material, Figure S1) were collected in a swampy boreal forest (64.4° N, 40.5° E; Primorsky district of the Arkhangelsk region, Russia) in September 2020. The identification of the botanical material was carried out according to the herbarium of the Northern (Arctic) Federal University. Whole plants were air-dried, ground in a cutting mill to a particle size of 0.5–1 mm, and stored in the dark at room temperature.

Two-stage pressurized liquid extraction (PLE) was used to isolate extractives from the plant biomass. At the first stage (extraction with hexane) lipophilic substances (lipids, fatty acids, carotenoids, chlorophyll derivatives), were removed to avoid interferences in NMR and HPLC-HRMS analysis. The second stage (extraction with 95% aqueous acetone) ensured an efficient extraction of the polyphenolic compounds. PLE was carried out on an ASE-350 accelerated solvent extraction system (Dionex, Sunnyvale, CA, USA) at a temperature of 100 °C and a pressure of 100 bar. A sample of dry plant material (10.0 g) was placed into a stainless steel extraction vessel and subjected to extraction in three 10 min cycles under nitrogen atmosphere at each stage. After the first stage, the residual hexane in the plant material was evaporated in a nitrogen stream. The resulting acetone extract was concentrated under vacuum in a rotary evaporator until almost all acetone was removed, then rapidly frozen with liquid nitrogen, and freeze-dried in a FreeZone Triad lyophilization system (Labconco, Kansas City, MO, USA). The yield of the dry acetone extract was 390 mg (3.9%).

### 2.3. NMR Spectroscopy

The dry acetone extract (50.0 mg) was dissolved in 550 μL of DMSO-*d*_6_ and subjected to 2D NMR analysis. The structures of individual compounds isolated from the extract by preparative HPLC were confirmed by ^1^H and 2D NMR spectra. The obtained fractions were dissolved in CD_3_OD and transferred to the respective NMR tubes.

The NMR spectra were registered on an AVANCE III NMR spectrometer (Bruker, Ettlingen, Germany), with an operational frequency of 600 MHz for protons. The experiment parameters to register the 2D (^1^H-^13^C) heteronuclear single-quantum correlation (HSQC) spectrum were as follows: temperature of 25 °C, spectrum window width of ~13 ppm for F2 and ~200 ppm for F1, with 1024 × 256 accumulations, and 8 scans. The delay time between pulses (D1) was 2.0 s. The experiment parameters for the 2D (^1^H-^13^C) heteronuclear multiple-bond correlation (HMBC) spectrum were as follows: temperature of 25 °C, spectrum window width of ~12 ppm for F2 and ~240 ppm for F1, with 2048 × 512 accumulations, 8 scans. The delay time between pulses (D1) was 2.0 s. The spectra were referenced to the residual DMSO peak at 39.5/2.51 ppm. For obtaining the ^1^H NMR spectra, the signals from the residual water present in the samples were suppressed using the standard water single-frequency pre-saturation pulse sequence (“zgesgp”) of the Topspin 3.2 instrument software package. The spectra were referenced to the residual CD_3_OD peak at 3.31 ppm.

The unknown screening and identification of organic compounds in the plant extract were carried out using ACD/Structure Elucidator expert system software (ACD/Labs, Toronto, ON, Canada) with the NMR spectra database and the available literature data [8]. The assignment of unknown peaks was carried out by two approaches. The first one was based on the use of the NMR chemical shift database and the correlations present in the diagnostic NMR spectra. The second approach involved constructing model spectra of the putative compounds using the HOSE-code algorithm and comparing the chemical shift values for the unknown cross-peaks with the data obtained from the model spectra.

### 2.4. Liquid Chromatography–high-Resolution Mass Spectrometry

An analysis of the obtained extract by liquid chromatography–high-resolution mass spectrometry was carried out on an HPLC-QTOF-HRMS system consisting of an LC-30 Nexera (Shimadzu, Kyoto, Japan) chromatograph with two LC-30AD pumps and a high-pressure gradient system, a DGU-20A5R vacuum degasser, an SIL-30AC autosampler, a CTO-20A column thermostat, and an SPD-M20A diode array UV-VIS spectrophotometric detector (DAD) combined with a TripleTOF 5600+ quadrupole time-of-flight (Q-TOF) mass spectrometer (ABSciex, Concord, ON, Canada) and equipped with a DuoSpray ion source with an electrospray ionization (ESI) probe.

Chromatographic separation was achieved on a Nucleodur PFP reversed-phase column (Macherey-Nagel, Duren, Germany), 2×150 mm, with a 1.8 µm particle-sized pentafluorophenylpropyl sorbent in gradient elution mode. The mobile phase consisted of water (A) and acetonitrile (B) containing 0.1% of formic acid. The flow rate was 0.3 mL min^–1^. The following gradient program was used: 0–3 min with 10% A, and 3–40 min ramped up to 40–45 min with 100% B. The column temperature was 40 °C, and the injection volume was 2 µL. Spectrophotometric detection was carried out in a wavelength range of 220–600 nm.

Mass spectrometry detection was carried out in the positive ion ESI mode. The ion source parameters were as follows: temperature—400 °C; the nebulizing, drying, and curtain gas pressure were 40, 40, and 30 psi, respectively; ESI capillary voltage—5500 V; declustering potential—80 V. The full scan mass spectra were recorded in a *m*/*z* range of 150–1500. Information-dependent acquisition (IDA) was used for obtaining the tandem (MS/MS) mass spectra of the detected compounds. The precursor ions with a signal intensity of > 100 cps were isolated by quadrupole and subjected to collision-induced dissociation (CID) using the following parameters: isolation window width—0.7 Da, collision gas—nitrogen, *m*/*z* range—20–1200, collision energy—50 eV, collision energy spread—30 eV. Mass scale calibration was performed in automatic mode immediately before each chromatographic run using sodium formate solution as a standard.

The mass spectrometry and chromatography system control, data collection, and processing were performed using the Analyst TF 1.8, PeakView, and Formula Finder software packages (ABSciex, Concord, ON, Canada). The elemental compositions of the analytes were determined based on their accurate masses, isotopic distributions, and MS/MS spectra applying the following constraints: maximum numbers of C, H, and O atoms—100, 200, and 50, respectively.

The quantification of the analytes in the obtained plant extract was carried out by HPLC-UV with a working wavelength of 280 nm corresponding to the absorption maximum of the target compounds. Fraction 3, with the highest purity, was used as an analytical standard for calibration curve construction.

### 2.5. Preparative Chromatography and Fraction Purity Assessment

The preparative HPLC separation was carried out on an LC-20 Prominence preparative HPLC system (Shimadzu, Kyoto, Japan) consisting of two LC-20AP pumps with a maximum flow rate of 150 mL min^–1^ each, a DGU-A5R vacuum degasser, a CTO-20A column thermostat with an integrated manual loop injector, an SPD-M20A diode array spectrophotometric detector with a preparative high-flow cell, and an FRC-10A fraction collector. The system was controlled with LabSolutions software (Shimadzu, Kyoto, Japan). Semi-preparative Nucleodur C18 Gravity (Macherey-Nagel, Duren, Germany), 250 × 21 mm, 5 µm particle size, with an octadecyl silica reversed stationary phase was used. The mobile phase flow rate was 21.0 mL min^–1^, column temperature—40 °C, and detection wavelength—280 nm. The mobile phase consisting of components A (0.1% aqueous solution of formic acid) and B (acetonitrile with 0.1% of formic acid) was supplied by separate HPLC pumps. The high-pressure gradient was programmed as follows: 0–20 min—35% B; 20–30 min—linear increase to 100% B, held for 10 min. The total separation time was 40 min.

The freeze-dried plant extract (100 mg) was dissolved in 2 mL of 50% aqueous methanol and centrifuged at 15,000 rpm, and then the whole volume was injected into the HPLC system. The fraction collection was performed according to the following program, optimized in the preliminary chromatographic runs and corresponding to the chromatographic peaks of six major phenolic compounds: F1—4.95–5.60 min, F2—9.30–10.30 min, F3—13.80–15.55 min, F4—20.55–22.90 min, F5—24.70–25.00 min, F6—30.27–30.45 min. The fractions obtained in the three consecutive chromatographic runs were combined and then evaporated under vacuum in a rotary evaporator to dryness, redissolved in 1 mL of methanol, and evaporated again to dryness under nitrogen stream in chromatographic vials.

The purity of the obtained fractions was estimated in an additional chromatographic analysis using the same analytical HPLC system with a spectrophotometric diode array detector and under chromatographic conditions, as described in Section 2.4. The content of the target compound (%) was calculated as a ratio of the target chromatographic peak area and the total area of all peaks on the chromatogram.

### 2.6. In Vitro Antioxidant Capacity Assay (PCL Assay)

The antioxidant activity (superoxide anion radical scavenging) of the plant extract and obtained fractions were determined by the photochemiluminescence (PCL) technique using a Photochem analyzer (Analytik Jena AG, Jena, Germany) according to the antioxidant capacity of the lipid-soluble (ACL) compound determination protocol [17]. A commercially available ACL reagent kit (Analytik Jena AG, Jena, Germany), including the Trolox standard, was used in the analysis procedure. The sample solution in methanol (20 µL) with a concentration of 50 µg mL^–1^ was mixed with ready-to-use ACL reagents according to the manufacturer’s manual and introduced in the analyzer. Calibration was performed immediately before analysis using the Trolox standard solutions in the methanol. The instrument control, data collection, and quantification were carried out using PCLSoft software (Analytik Jena AG, Jena, Germany). The results (mmol g^–1^) were calculated on the basis of the Trolox equivalents (TEs). All assays were performed in triplicate, with further standard deviation calculations.

### 2.7. In Silico Biological Activity/Toxicity Assessment

The acute toxicity, mutagenicity, health effects, and absorption, distribution, metabolism, and excretion (ADME) parameters were predicted with ACD/Labs Percepta Platform software v. 2021.1.3 (Advanced Chemistry Development, Inc., Toronto, ON, Canada) using quantitative structure–activity/toxicity relationship (QSAR/QSTR) algorithms.

## 3. Results and Discussion

### 3.1. Two-Dimensional NMR Screening of Plant Extract Chemical Composition

For the primary screening of the chemical composition of the *P. commune* acetone extract, the combination of the two-dimensional NMR spectroscopy and the computational expert system for processing the obtained data, which was proven in a recent study of polyphenolic compounds in birch phloem [18], was used. This made it possible to isolate the signals of individual components of a complex mixture and presumably identify them on the basis of a library search.

**Figure 1:**
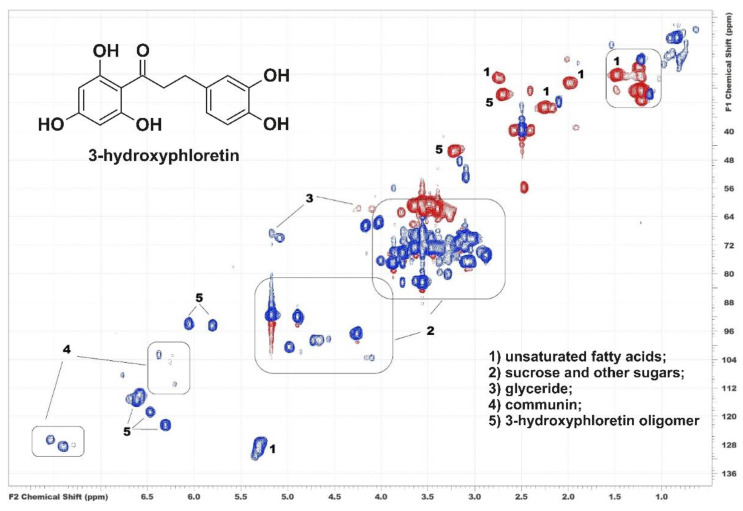
^1^H-^13^C HSQC NMR spectrum of acetone extract of *P. commune* and assignment of the main cross-peaks.

An analysis of the HSQC (^1^H-^13^C) NMR spectrum of the extract (Figure 1) revealed the presence of fatty acids, including their triglycerides, various sugars (among which sucrose predominated), and a flavanone derivative tentatively identified as communin A (PubChem ID 44233686). This is in good agreement with the literature data since these compounds were previously considered the main components of moss extracts [8,10]. Special attention must be drawn to the aromatic region of the spectrum, in which additional intense cross-peaks attributed to the structure of dihydrochalcone were observed. In particular, four cross-peaks resonating from the aromatic CH-groups in the structure of the pyrocatechol unit and two cross-peaks from the aromatic CH-groups in the structures of the phloroglucinol unit were detected in the regions of δc/δ_H_ 112–125/6.0–7.0 and 90–100/5.7–6.2 ppm, respectively. The key correlations between the described cross-peaks and signals of the methylene groups in the ^1^H-^13^C HMBC spectrum at δc/δ_H_ 47.4/3.32 and 31.7/2.83 ppm (Figure S2) show that they may belong to the structure of 3-hydroxyphloretin (3-(3,4-dihydroxyphenyl)-1-(2,4,6-trihydroxyphenyl)propan-1-one). It is worth noting that according to the key correlation in the ^1^H-^13^C HMBC spectrum from H-6 to C-3″, the 3-hydroxyphloretin structure, probably belongs to dimer or polymer formed through a carbon–carbon bond between the phloroglucinol unit of the first molecule and the pyrocatechol unit of the second one (“head-to-tail” coupling). However, HSQC and HMBC NMR spectra do not allow for unambiguous determination of the polymerization degree, and identification of the components of a complex mixture only by 2D NMR data cannot be considered sufficiently reliable due to the significant overlapping of the cross-peaks of various compounds. In this regard, further research was focused on a more detailed study of the structure of the detected compounds using liquid chromatography and high-resolution mass spectrometry techniques complementary to NMR.

### 3.2. HPLC-DAD-HRMS Analysis of Phenolic Compounds in the Plant Extract

**Figure 2:**
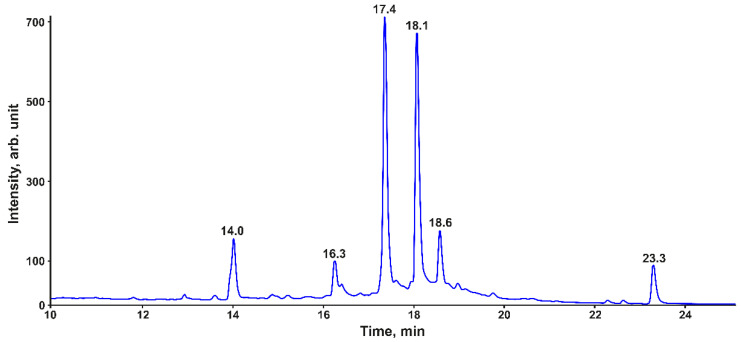
HPLC-UV (280 nm) chromatogram of acetone extract of *P. commune*.

Since phenolic compounds are characterized by intense absorption in the UV region of the spectrum, their selective detection and identification can be most effectively carried out by combining spectrophotometric and mass spectrometric detection [19]. Figure 2 shows the HPLC-UV chromatogram of *P. commune* acetone extract at a detection wavelength of 280 nm, corresponding to the supposed position of the absorption maximum of 3-hydroxyphloretin and other substituted phenols. The most intense signals were observed for the components for which the retention time (t_R_) was in the range of 10–25 min, while the total number of detected chromatographic peaks reached several tens. Among them, the peaks of the six major compounds dominated, the absorption spectra of which had a maximum in the wavelength range of 270–290 nm.

The compound with a retention time of 23.3 min showed a signal of the protonated molecule ([M + H]^+^) at *m*/*z* 343.1339 in the ESI+ high-resolution mass spectrum, which corresponds to the elemental composition C_23_H_18_O_3_ (Δ*m*/*z* = 3.0 ppm). Based on the MS/MS (CID) and NMR spectra, this analyte was assigned to communin A, which was previously found in *P. commune* and described in the literature [10].

Another secondary metabolite eluted at 14.0 min had the elemental composition C_46_H_34_O_16_ ([M + H]^+^ *m*/*z* 843.1942, Δ*m*/*z* = 2.7 ppm). The MS/MS spectrum of this compound demonstrates the sequential elimination of two C_9_H_6_O_4_ neutral fragments, which, according to the literature data [1], can be attributed to coumarins commonly found in mosses. Thus, this metabolite can be considered a tetrameric polyphenol with two coumarin moieties in its structure. Due to the rather high molecular weight and the complexity of the tandem mass spectrum, the exact structure of this compound has not yet been established.

Of greatest interest is a group of four peaks with retention times of 16.3, 17.4, 18.1, and 18.6 min, which accounted for most of the total area of all peaks in the chromatogram and had the identical UV-spectra with λ_max_ = 289 nm and a shoulder at ~330 nm (Supplementary Material, Figure S3). They belong to structurally similar compounds that differ from each other by a mass of 288.0640 Da, corresponding to a fragment with the elemental composition C_15_H_12_O_6_. Based on the data obtained by 2D-NMR (Section 3.1), it can be attributed to the 3-hydroxyphloretin (C_15_H_14_O_6_) residue (loss of two hydrogen atoms for the formation of a C-C bond with neighbor units) in the set of oligomers with a degree of polymerization from 2 to 5 (Table 1).

**Figure 3:**
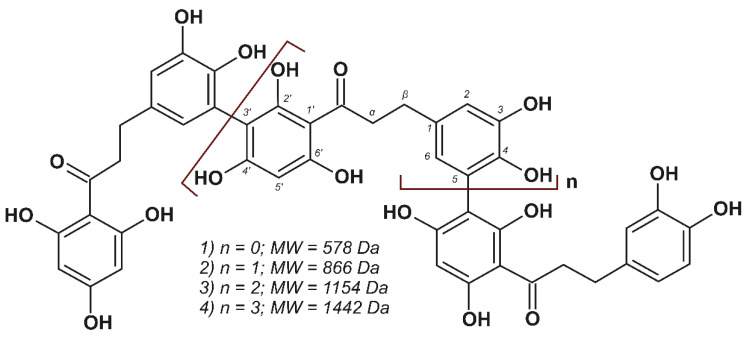
Chemical structure of 3-hydroxyphloretin oligomers listed in Table 1.

The tandem mass spectra of these compounds, with the exception of the analyte with *m*/*z* 1443.342, which cannot be isolated by a quadrupole mass filter, are shown in the Supplementary Material, Figures S4–S6. They contain similar sets of fragment ions and provide additional structural information to confirm the conclusions about their nature. In the course of CID, the loss of up to two water molecules by the precursor ion was observed and apparently associated with the elimination of the hydroxyl group in the alpha position of the alkyl chain, which was formed as a result of keto-enol tautomerism. The most intense signals in the MS/MS spectra observed at *m*/*z* 289.0709 belong to the 3-hydroxyphloretin residue ([M – H]^+^), which was formed upon heterolytic cleavage of the aryl–aryl bond between monomeric units. Accordingly, there was also a loss of 3-hydroxyphloretin neutral molecule C_15_H_14_O_6_ (290.0805 Da) by the precursor ion. In the low molecular weight region, the signals of the product ions formed during the CID of 3-hydroxyphloretin were observed, while the final aromatic product was a fragment ion of 4-methylcatechol ([M – H]^+^) with *m*/*z* 123.0441. Thus, combining the results of the NMR and HPLC-HRMS analyses, the structure of the compounds listed in Table 1 is represented in Figure 3.

The discovery of 3-hydroxyphloretin-derived oligomeric dihydrochalcones in *P. commune* is of great interest since such compounds have not yet been found in moss extracts. Moreover, dihydrochalcones have mainly been found as monomeric structures [20,21], and to date, only one representative of naturally occurring bisdihydrochalcones (Pierotin A) isolated from the leaves of Japanese pieris (*Pieris japonica*) belonging to *Ericaceae* family is known [22].

It is worth noting that the detection of the oligomers presented in Figure 3 can also shed light on the nature of lignin-like biopolymers in *P. commune*, which have been previously reported in the literature [15,16]. It can be assumed that the acetone extraction used in our study made it possible to extract only a low molecular weight fraction of the polymeric dihydrochalcone synthesized by the plant by analogy with algal phlorotannins [23] with the formation of fucol-type aryl–aryl bonds. This issue requires additional study and is beyond the scope of the present work.

### 3.3. Isolation and Characterization of Individual Dihydrochalcones

Since the components of the studied extract differ significantly in polarity, reverse-phase HPLC was chosen for the preparative isolation of individual phenolic compounds. Although a more available octadecyl silica sorbent, differing from that in analytical separations, was used as a stationary phase in the preparative column, the elution order of the analytes did not change (Supplementary Material, Figure S7). The achieved chromatographic resolution ensured an acceptable separation of the components even under conditions of a significant overload of the column (injection of 100 mg of extract), which is typical for the preparative HPLC. This approach allowed for the obtainment of six fractions (F1–F6) suitable for further characterization by NMR and other analytical techniques. Fractions F2–F5, representing the studied 3-hydroxyphloretin oligomers, had the following characteristics:

F2: 3-hydroxyploretin dimer. Light beige powder; purity: 89%; UV (MeOH): λ_max_ (log ε) 221 (0.337), 289 (0.234) nm; ^1^H NMR (600 MHz, CD_3_OD) and ^13^C NMR (150 MHz, CD_3_OD): Table 2; HRMS (ESI+): *m*/*z* 579.1505 ([M + H]^+^), calcd. for C_30_H_26_O_12_, 579.1499, Δ*m*/*z =* 1.0 ppm).

F3: 3-hydroxyploretin trimer. Light beige powder; purity: 97%; UV (MeOH): λ_max_ (log ε) 221 (0.345), 289 (0.256) nm; ^1^H NMR (600 MHz, CD_3_OD) and ^13^C NMR (150 MHz, CD_3_OD): Table 2; HRMS (ESI+): *m*/*z* 867.2149 ([M + H]^+^), calcd. for C_45_H_38_O_18_, 867.2131, Δ*m*/*z =* 2.1 ppm).

F4: 3-hydroxyploretin tetramer. Light beige powder; purity: 85%; UV (MeOH): λ_max_ (log ε) 221 (0.344), 289 (0.284) nm; ^1^H NMR (600 MHz, CD_3_OD) and ^13^C NMR (150 MHz, CD_3_OD): Table 2; HRMS (ESI+): *m*/*z* 1155.278 ([M + H]^+^), calcd. for C_60_H_50_O_24_, 1155.277, Δ*m*/*z =* 0.9 ppm).

F5: 3-hydroxyploretin pentamer. Light beige powder; purity: 71%; UV (MeOH): λ_max_ (log ε) 221 (0.190), 289 (0.175) nm; ^1^H NMR (600 MHz, CD_3_OD) and ^13^C NMR (150 MHz, CD_3_OD): Table 2; HRMS (ESI+): *m*/*z* 1443.342 ([M + H]^+^), calcd. for C_75_H_62_O_30_, 1443.340, Δ*m*/*z =* 1.4 ppm).

Fraction F3 (trimer) had the highest purity (97%) due to the best separation from other compounds (Supplementary Material, Figure S7) and the highest content in the plant extract. This made it possible to use fraction F3 as an analytical standard for the quantitative determination of 3-hydroxyphloretin oligomers in the moss extract by HPLC-DAD, considering their close absorption coefficients. The obtained value was 70 mg g^–1^ or 7% by weight of all compounds extracted with 95% acetone under PLE conditions.

An analysis of the ^1^H (Supplementary Material, Figures S8–S11) and 2D NMR (Supplementary Material, Figures S12 and S13) spectra of the obtained individual compounds (Table 2) made it possible to confirm the structure of the 3-hydroxyphloretin oligomers presented in Figure 3. This is evidenced by the same set of signals in the ^1^H spectra, which show two distinct signals of aromatic protons of the phloroglucinol unit at δ_H_ 5.82 and 6.03, a multiplet of aromatic protons of the catechol unit at δ_H_ 6.5–6.8, and a broad signal of the methylene group at δ_H_ 2.83. The integral intensities of the signals in the ^1^H NMR spectra confirm that the mass of the oligomers differed by one 3-hydroxyphloretin unit. The spectral patterns in the HSQC and HMBC NMR spectra are also consistent with the proposed structural formulas.

Fraction F1 corresponded to the compound with *m*/*z* 843.1942 [M + H]^+^ and a retention time of 14.0 min in the HPLC-DAD-HRMS analysis (Figure 2). Although its purity was 81%, it was not possible to unambiguously establish the structure of the main component based on the NMR spectrum, which may have been due to the interferences from impurities. The obtained NMR spectra of fraction F6 (81% purity) confirm that its major constituent is communin A (Section 3.2).

### 3.4. Antioxidant Activity and Estimated Health Impact

The polyphenolic nature of the identified dihydrochalcones determines their antioxidant properties. The most important of them seems to be their free radical scavenging ability, protecting DNA from oxidative damage resulting from the attack of reactive oxygen species (**^·^**OH, H_2_O_2_, and O_2_**^·^**^–^) on the DNA oligonucleotides [24]. To assess the protective effect of the isolated 3-hydroxyphloretin oligomers, the obtained fractions F2–F5 and the initial acetone extract were studied by the photochemiluminescence (PCL) method, involving the UV-induced generation of the superoxide anion radicals (O_2_**^·^**^–^), their interaction with a luminescent compound with visible light emission, and the detection of luminescence quenching upon the addition of the tested compound.

**Figure 4:**
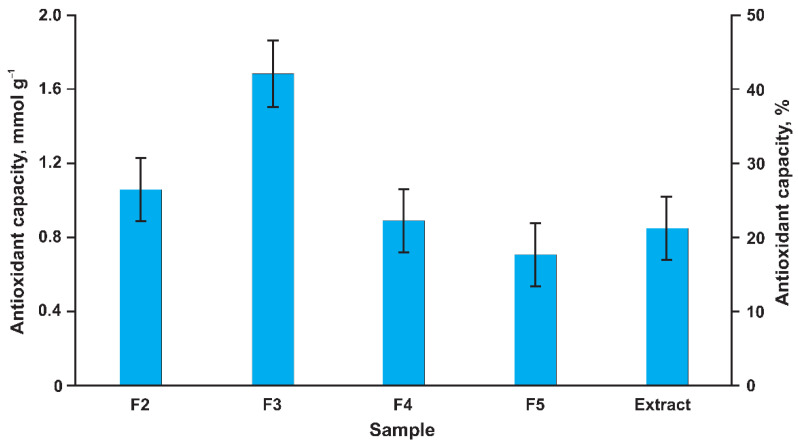
Results of in vitro antioxidant capacity assay by PCL method (in Trolox equivalent).

The obtained results expressed in the Trolox equivalent (Figure 4) demonstrate a high antioxidant capacity of all the studied samples. The highest value was observed for fraction F3 (3-hydroxyphloretin trimer), which may have been due to the highest content of oligomeric dihydrochalcone. It amounted to 1.7 mmol g^–1^ and corresponded to 425 mg of Trolox per 1 g of the sample. The antioxidant activities of the other fractions and the extract as a whole were 1.5–2 times lower. This suggests that the oligomers of 3-hydroxyphloretin make a decisive contribution to the antioxidant properties of *P. commune* extract and are of high value as a promising raw material for the production of biologically active additives or pharmaceuticals with antioxidant action.

An assessment of acute toxicity and mutagenicity, carried out by the QSAR/QSTR approach on an example of 3-hydroxyphloretin trimer, showed low danger to the human body; the predicted LD_50_ values were in the range of 140–9900 mg kg^–1^, and the probability of a positive Ames test was 0.13. Physico-chemical and ADME profiling revealed high lipophilicity (Log*P* = 5.56), poor cell permeability, and the highest (100%) plasma protein binding (PPB) ability of this polyphenolic compound (Supplementary Materials, Protocol of QSAR/QSTR testing). It is known that various derivatives of dihydrochalcones possess a wide range of biological activities [25] in addition to antioxidant properties. For this reason, 3-hydroxyphloretin oligomers from *P. commune* deserve close attention in pharmaceutical development and require further research on their properties.

## 4. Conclusions

The low molecular weight polyphenolic fraction of the moss *Politrichum commune*, obtained by pressurized liquid extraction with aqueous acetone, contains substantial amounts (~7%) of dihydrochalcone derivatives that have not been previously found in this plant and can be considered as dominant aromatic components along with the known flavanone–styryl compound communin. They are represented by 3-hydroxyphloretin oligomers with a polymerization degree of 2–5, among which trimer and tetramer predominate. The isolated individual oligomers are characterized by high antioxidant activity (free radical scavenging) ranging from 17.5 to 42.5 % with respect to Trolox, as well as low toxicity, which makes them a promising source for the production of biologically active food additives and pharmaceuticals. The possible presence of 3-hydroxyphloretin oligomers with a higher degree of polymerization in the moss biomass may explain the nature of lignin-like polymers in *P. commune* described in the literature.

## Figures and Tables

**Table 1:** Major compounds and their tentative identification by HPLC-ESI(+)-HRMS.

**No**	Compound	t_R_, (min)	*m*/*z* [M + H]^+^	Elemental Composition	∆, ppm
1	3-hydroxyphloretin dimer	16.3	579.1505	C_30_H_26_O_12_	1.4
2	3-hydroxyphloretin trimer	17.4	867.2144	C_45_H_38_O_18_	1.5
3	3-hydroxyphloretin tetramer	18.1	1155.278	C_60_H_50_O_24_	1.7
4	3-hydroxyphloretin pentamer	18.6	1443.342	C_75_H_62_O_30_	1.6

**Table 2:** NMR Spectroscopic data for 3-hydroxyphloretin oligomers in fractions F2–F5.

No		δC *, ppm	δH, ppm (J, Hz)	HMBC Correlation
End groups				
1	qC	134.7	-	
2	CH	116.4	6.69, (m, overlapped)	1, 3, 6, β
3	qC	145.9	-	
4	qC	144.0	-	
5	CH	ND	ND	
6	CH	120.5	6.57, (m, overlapped)	2, 4, β
				
1′	qC	105.0	-	
2′	qC	165.8	-	
3′	CH	95.7	5.82, s	1′, 2′, 5′, 6′
4′	qC	168.9	-	
5′	CH	95.7	5.82, s	1′, 2′ 3′, 6′
6′	qC	165.8	-	
C=O		206.7	-	
α	CH_2_	47.4	3.32, t (overlapped)	β, C=O, 1
β	CH_2_	31.7	2.83, t (overlapped)	α, C=O, 1, 2, 6
Conjugated links				
1	qC	134.7	-	
2	CH	115.7	6.73, (m, overlapped)	1, 3, 6, β
3	qC	146.8	-	
4	qC	142.5	-	
5	qC	ND	-	
6	CH	124.3	6.54, (m, overlapped)	2, 4, β, 3′
1′	qC	105.8	-	
2′	qC	163.5 **	-	
3′	qC	106.2	-	
4′	qC	ND	-	
5′	CH	95.6	6.03, s	1′, 2′ 3′, 6′
6′	qC	163.5 **	-	
C=O		206.7	-	
α	CH_2_	47.2	3.37, t (overlapped)	β, C=O, 1
β	CH_2_	31.8	2.87, t (overlapped)	α, C=O, 1, 2, 6

* Chemical shifts based on HSQC and HMBC correlation peaks. ** Assignments for these carbons may be interchangeable.

## Data Availability

The data presented in this study are available in the article and Supplementary Materials.

## Supplementary Materials

The following supporting information can be downloaded at: https://www.mdpi.com/article/10.3390/metabo12100974/s1, Figure S1: *Polytrichum commune* at the collection site; Figure S2: Two-dimensional (^1^H-^13^C HMBC) NMR spectrum of *Polytrichum commune* extract (*a*) and main correlation of 3-hydroxyphloretin dimer (*b*); Figure S3: UV-VIS absorption spectra of 3-hydroxyphloretin oligomers extracted from HPLC-DAD chromatogram (at the maxima of the corresponding chromatographic peaks); Figure S4: Tandem mass spectrum of the dimer precursor ion with *m*/*z* 579.1514; Figure S5: Tandem mass spectrum of the trimer precursor ion with *m*/*z* 867.2183; Figure S6: Tandem mass spectrum of the tetramer precursor ion with *m*/*z* 1155.2786; Figure S7: Preparative HPLC-DAD (280 nm) chromatogram of *P. commune* acetone extract and F1–F6 fraction collection zones; Figure S8: ^1^H NMR spectrum of fraction F2; Figure S9: ^1^H NMR spectrum of fraction F3; Figure S10: ^1^H NMR spectrum of fraction F4; Figure S11: ^1^H NMR spectrum of fraction F5; Figure S12: Two-dimensional ^1^H-^13^C HSQC NMR spectrum of fraction F3; Figure S13: Two-dimensional ^1^H-^13^C HMBC NMR spectrum of fraction F3; QSAR/QSTR testing protocol of the 3-hydroxyphloretin trimer.
