# Supplementary material for: New Oligomeric Dihydrochalcones in the Moss Polytrichum commune: Identification, Isolation, and Antioxidant Activity
Source: Metabolites. 2022 Oct 14;12(10):974. doi: 10.3390/metabo12100974 (PMC9607112; doi:10.3390/metabo12100974)
Supplement: Supplementary file 1 [file metabolites-12-00974-s001.zip › metabolites-1962685-supplementary.pdf]

Supplementary material

New oligomeric dichydrochalcones in the moss Polytrichum commune: identification, isolation, and antioxidant activity

Contents:

Figure S1. *Polytrichum commune* at the collection site.

Figure S2. Two-dimensional (^{1}H-^{13}C HMBC) NMR spectrum of *Polytrichum commune* extract *(a)* and main correlation of 3-hydroxyphloretin dimer *(b)*.

Figure S3. UV-VIS absorption spectra of 3-hydroxyphloretin oligomers extracted from HPLC-DAD chromatogram (at the maxima of the corresponding chromatographic peaks).

Figure S4. Tandem mass spectrum of the dimer precursor ion with *m/z* 579.1514.

Figure S5. Tandem mass spectrum of the trimer precursor ion with *m/z* 867.2183.

Figure S6. Tandem mass spectrum of the tetramer precursor ion with *m/z* 1155.2786.

Figure S7. Preparative HPLC-DAD (280 nm) chromatogram of *P. commune* acetone extract and F1–F6 fraction collection zones.

Figure S8. ^{1}H NMR spectrum of fraction F2.

Figure S9. ^{1}H NMR spectrum of fraction F3.

Figure S10. ^{1}H NMR spectrum of fraction F4.

Figure S11. ^{1}H NMR spectrum of fraction F5.

Figure S12. Two-dimensional ^{1}H-^{13}C HSQC NMR spectrum of fraction F3**.**

Figure S13. Two-dimensional ^{1}H-^{13}C HMBC NMR spectrum of fraction F3**.**

Protocol of QSAR/QSTR testing the 3-hydroxyphloretin trimer (predicted with the ACD/Labs Percepta software v. 2021.1.3, Advanced Chemistry Development, Toronto, ON, Canada).

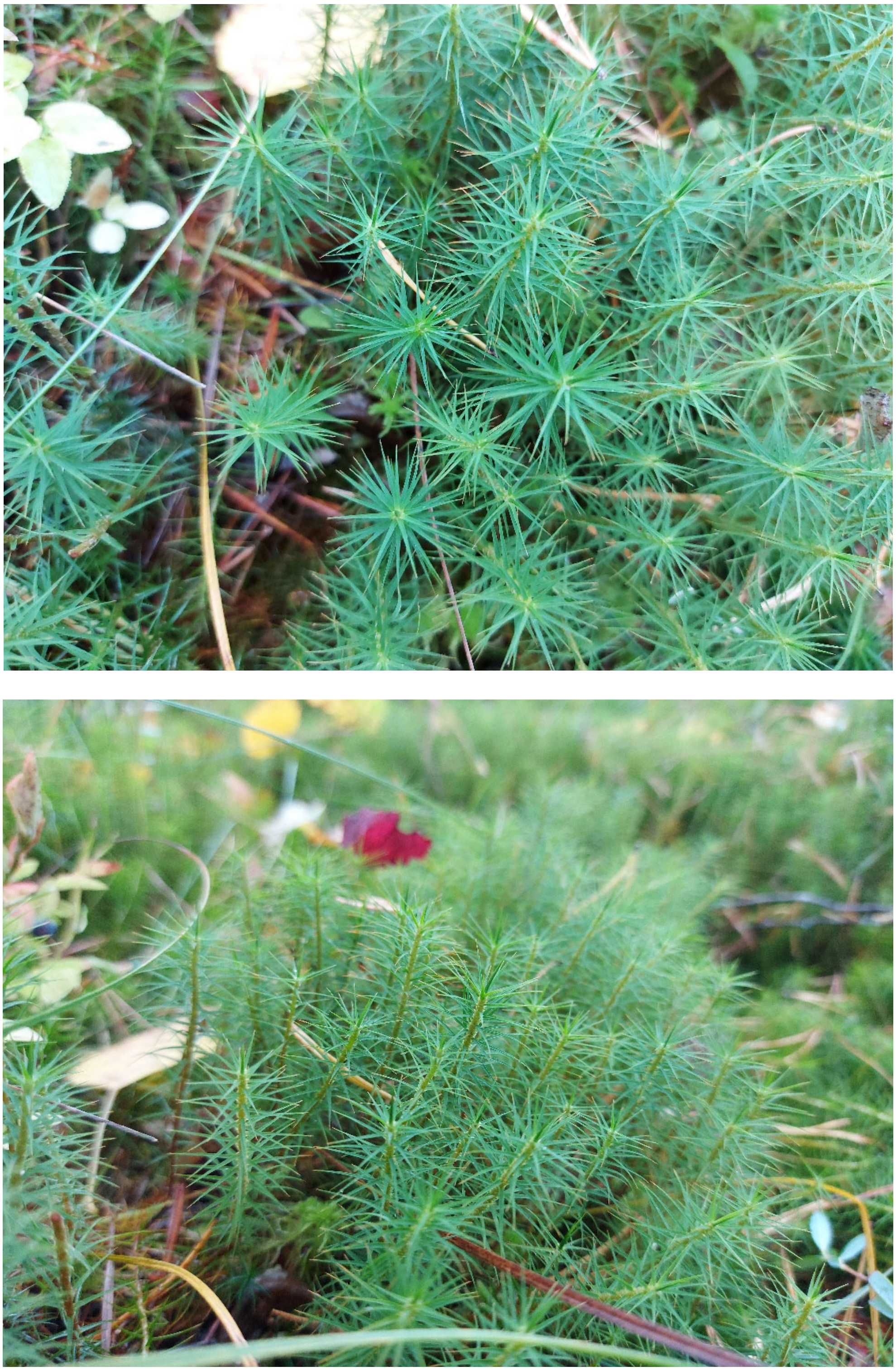

Figure S1. *Polytrichum commune* at the collection site.

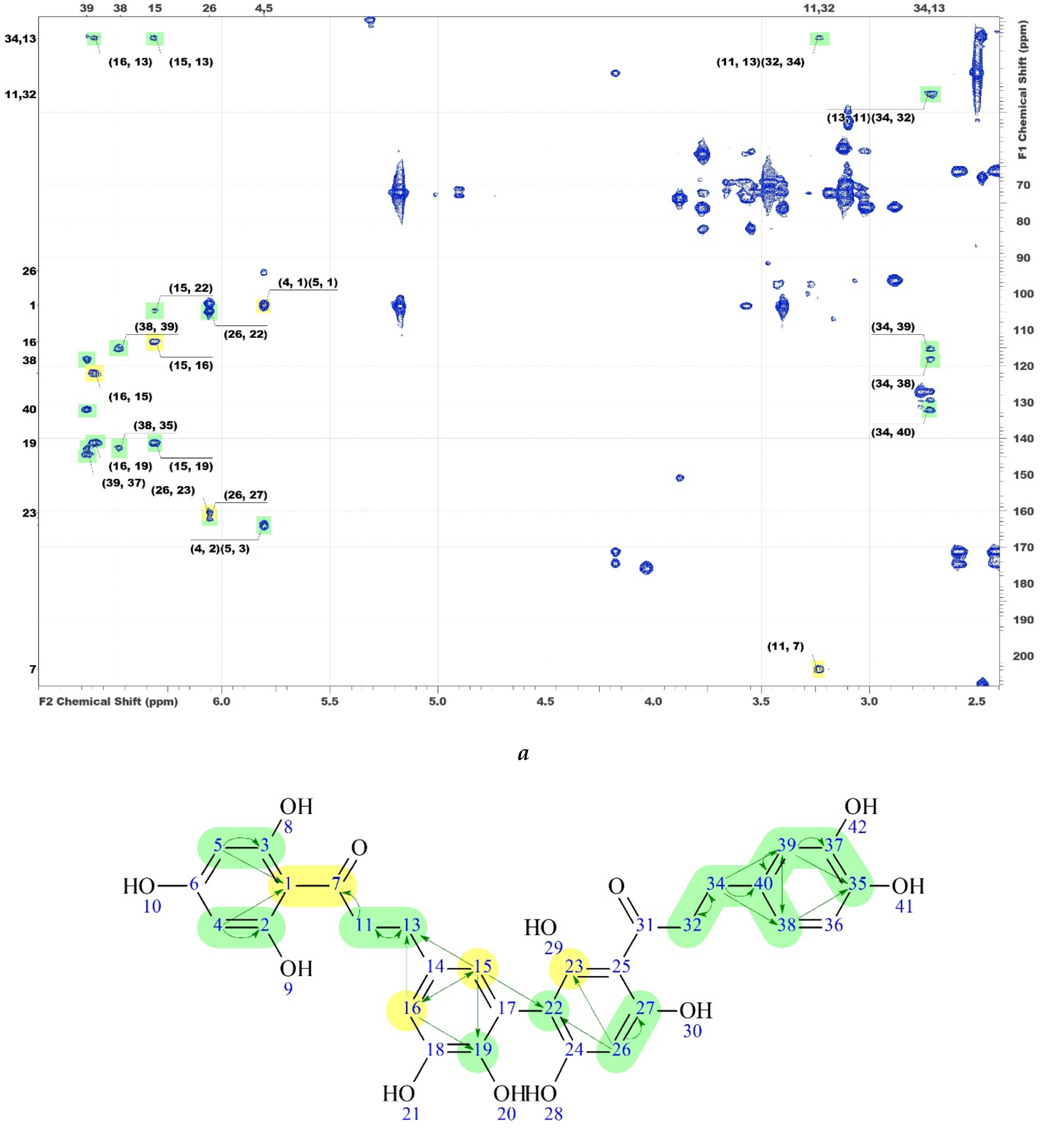

a

b

Figure S2. Two-dimensional (^{1}H-^{13}C HMBC) NMR spectrum of *Polytrichum commune* extract *(a)* and main correlation of 3-hydroxyphloretin dimer *(b)*. Colors designate the detected structural fragments and corresponding signals in the spectrum.

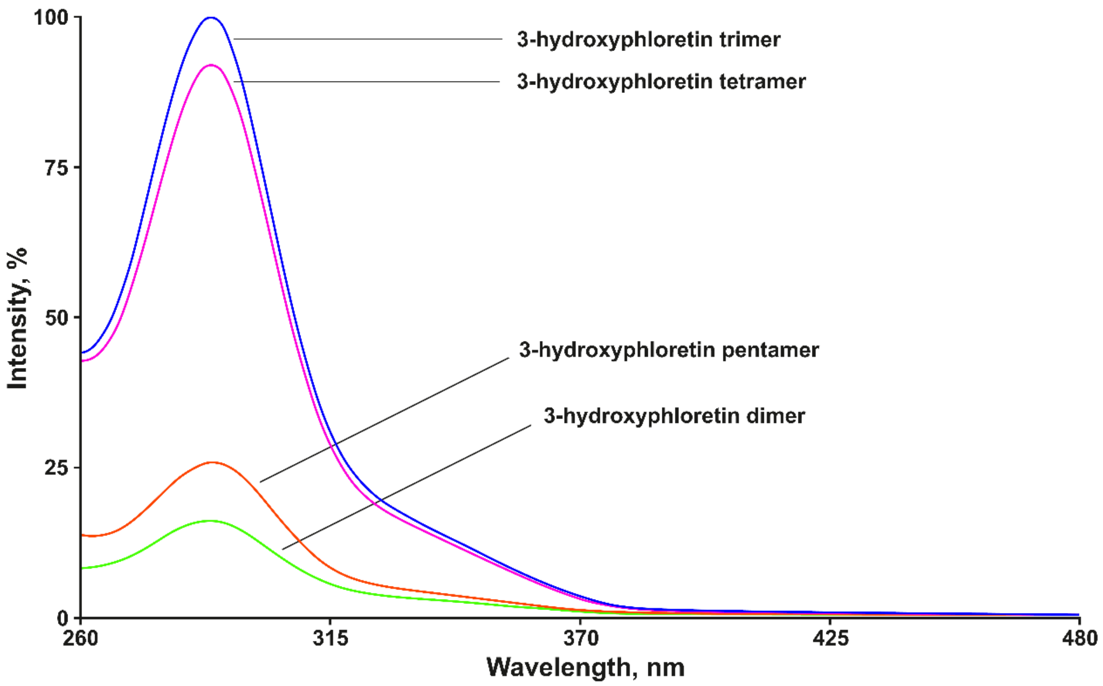

Figure S3. UV-VIS absorption spectra of 3-hydroxyphloretin oligomers extracted from HPLC-DAD chromatogram (at the maxima of the corresponding chromatographic peaks).

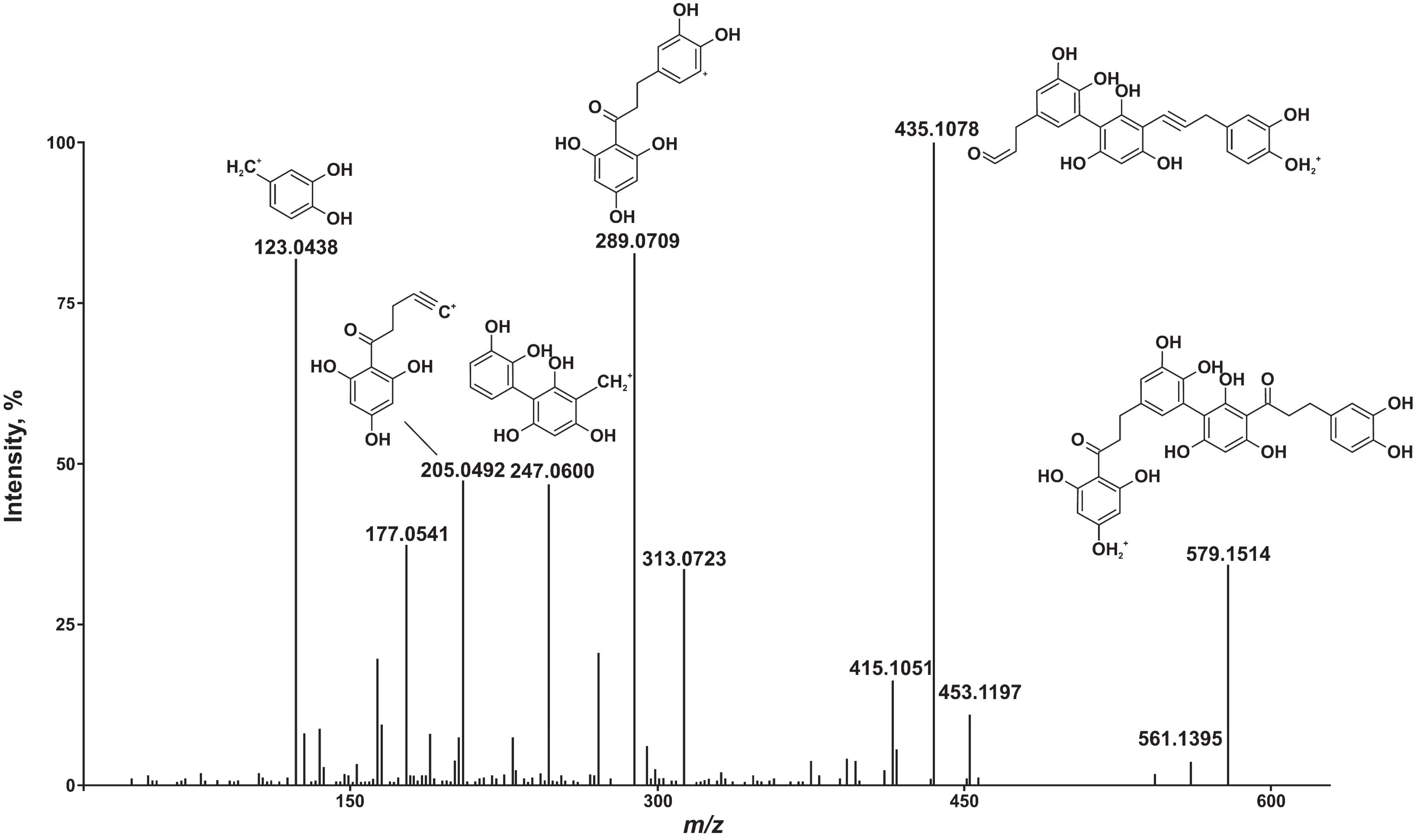

Figure S4. Tandem mass spectrum of the dimer precursor ion with *m*/*z* 579.1514.

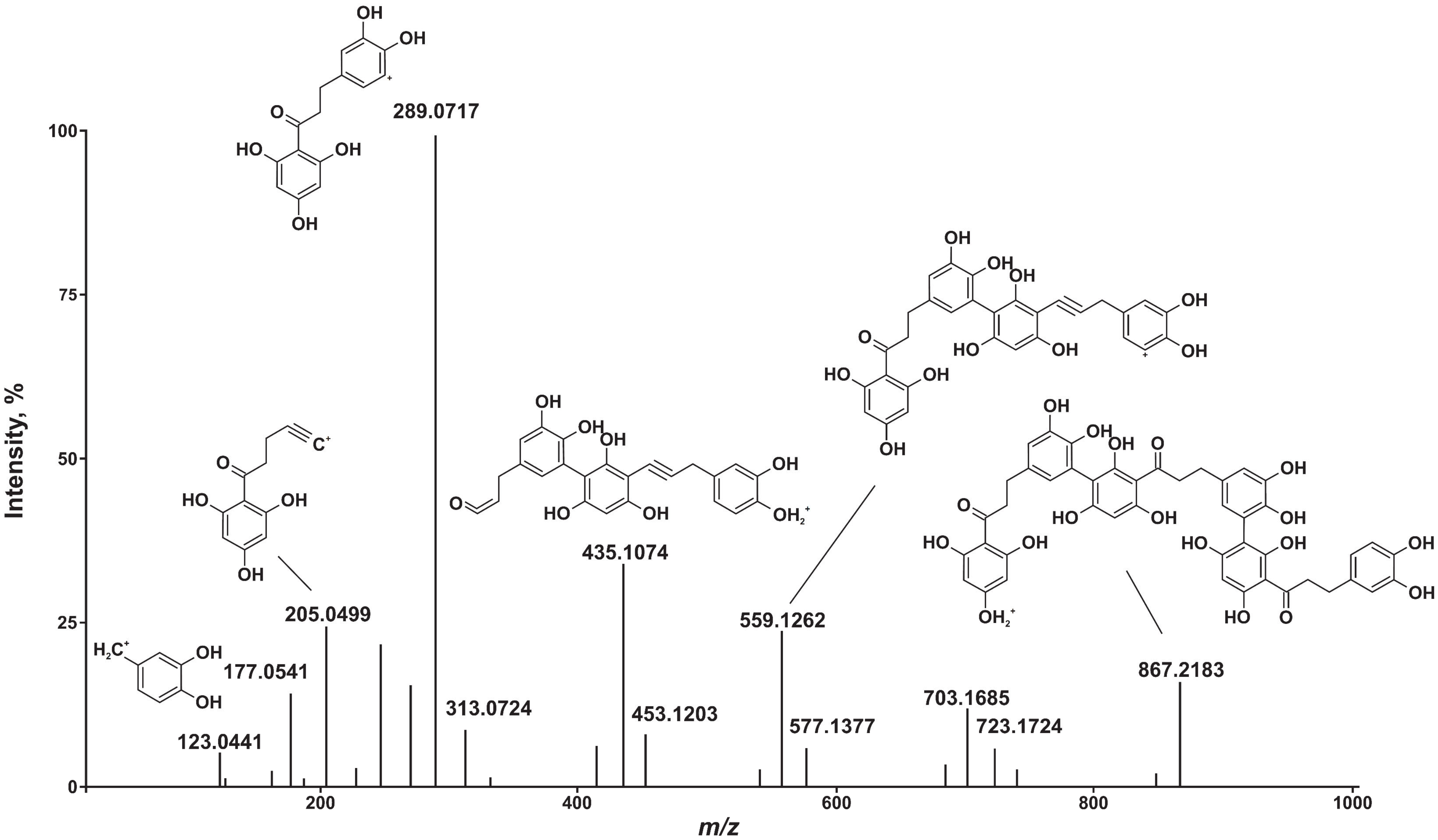

Figure S5. Tandem mass spectrum of the trimer precursor ion with *m*/*z* 867.2183.

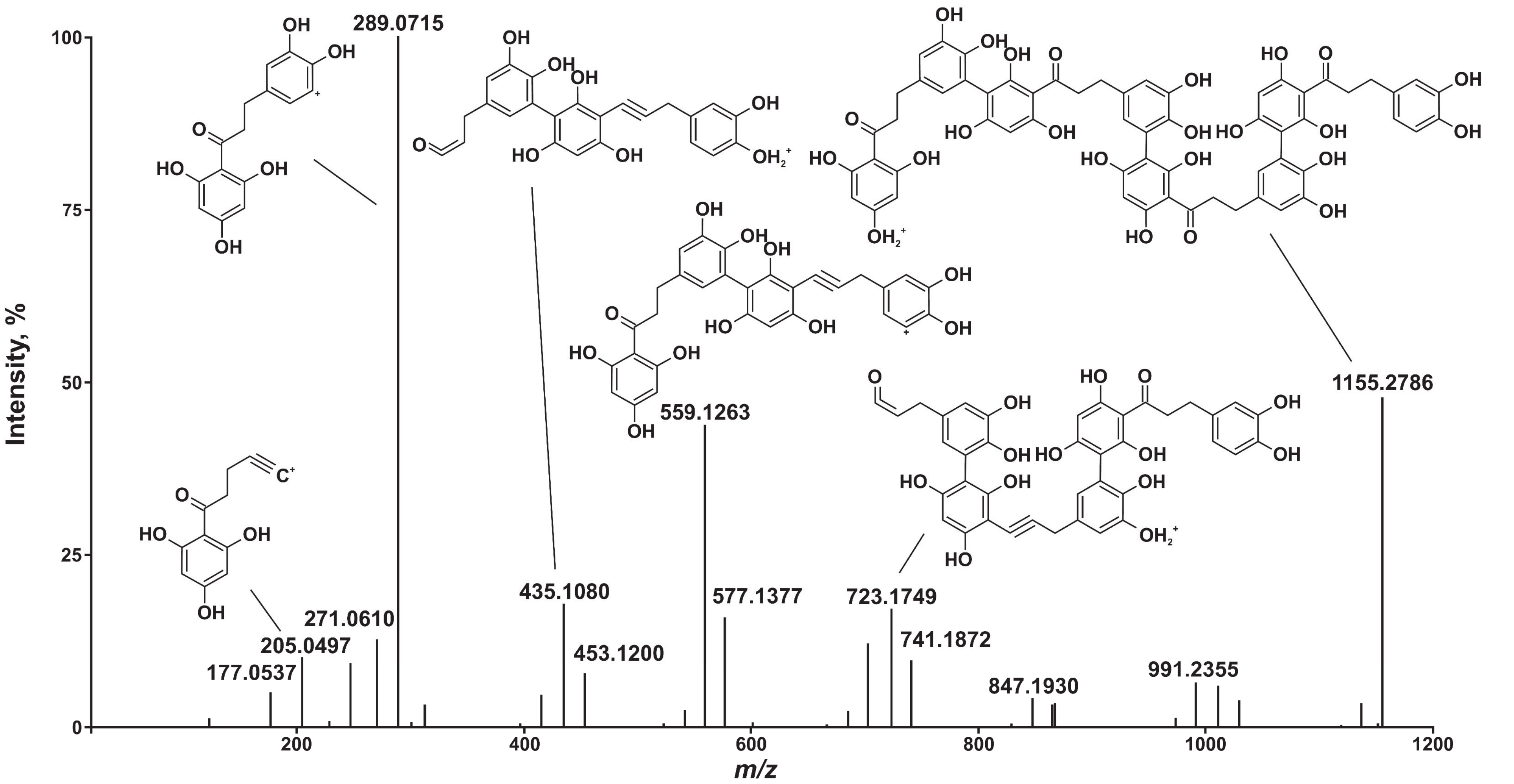

Figure S6. Tandem mass spectrum of the tetramer precursor ion with *m*/*z* 1155.2786.

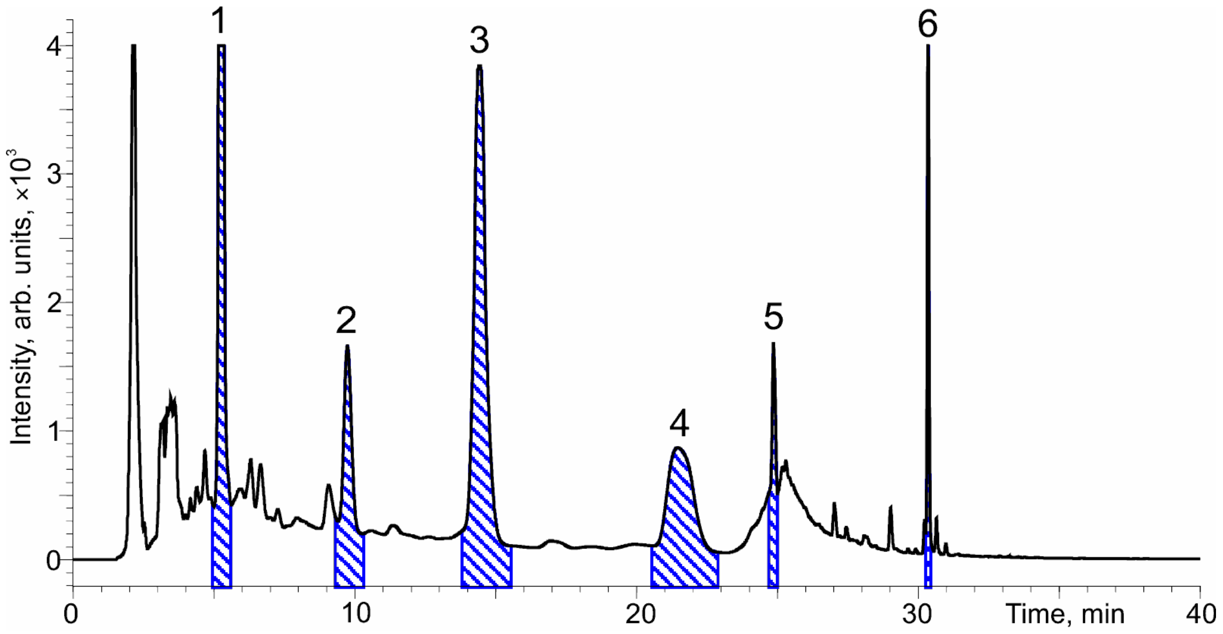

Figure S7. Preparative HPLC-DAD (280 nm) chromatogram of *P. commune* acetone extract and F1–F6 fraction collection zones.

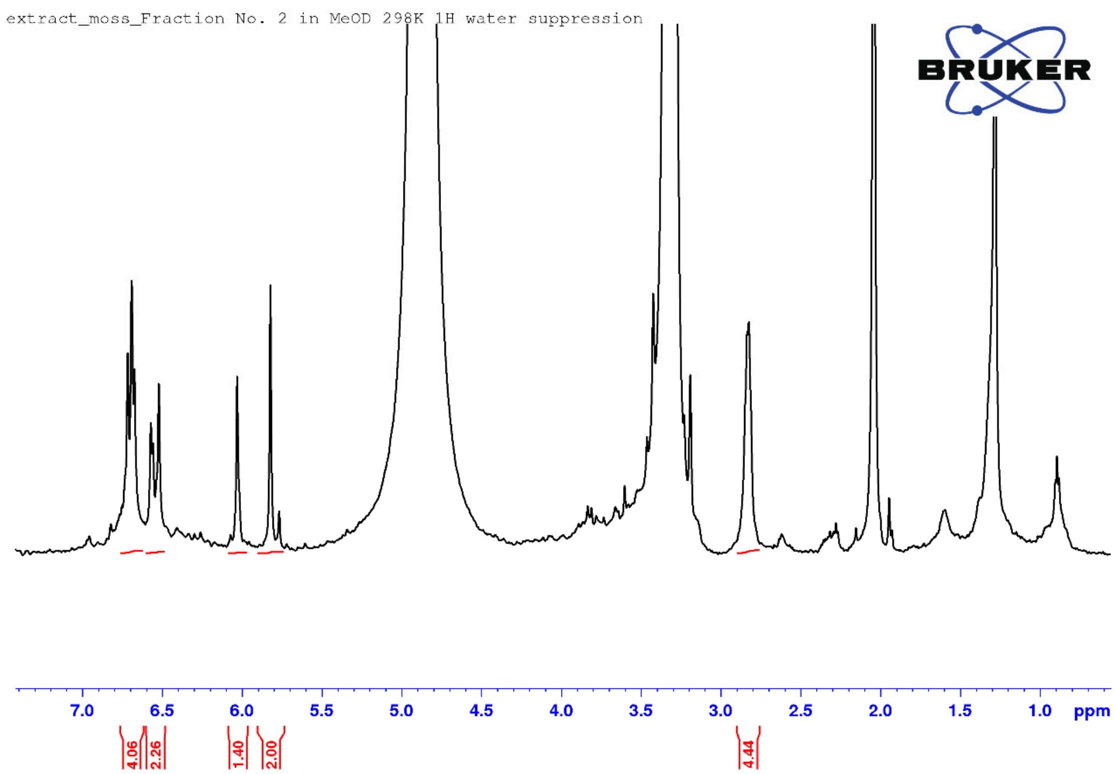

Figure S8. ^{1}H NMR spectrum of fraction F2.

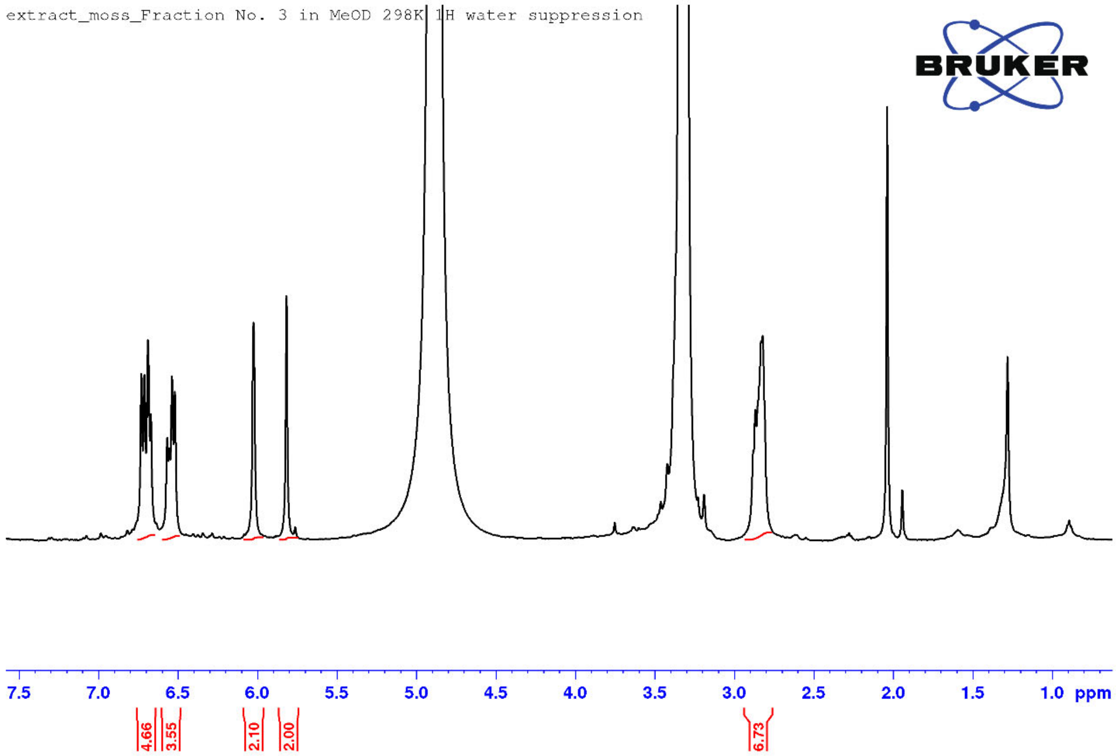

Figure S9. ^{1}H NMR spectrum of fraction F3.

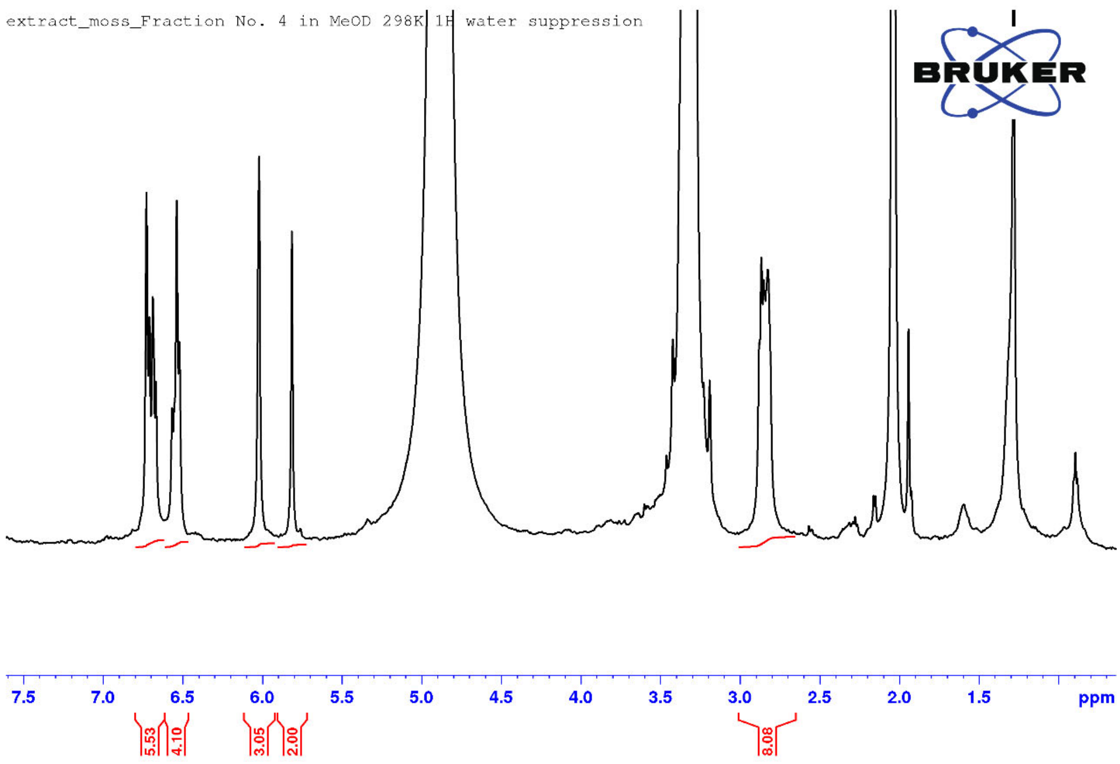

Figure S10. ^{1}H NMR spectrum of fraction F4.

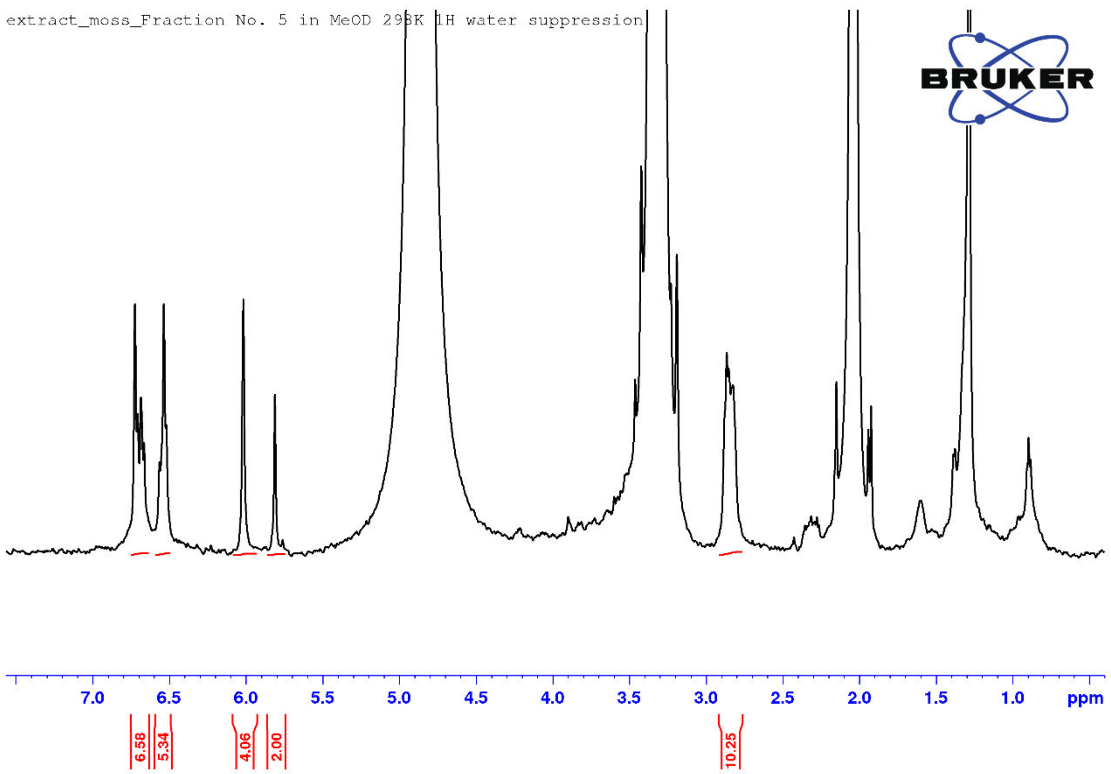

Figure S11. ^{1}H NMR spectrum of fraction F5.

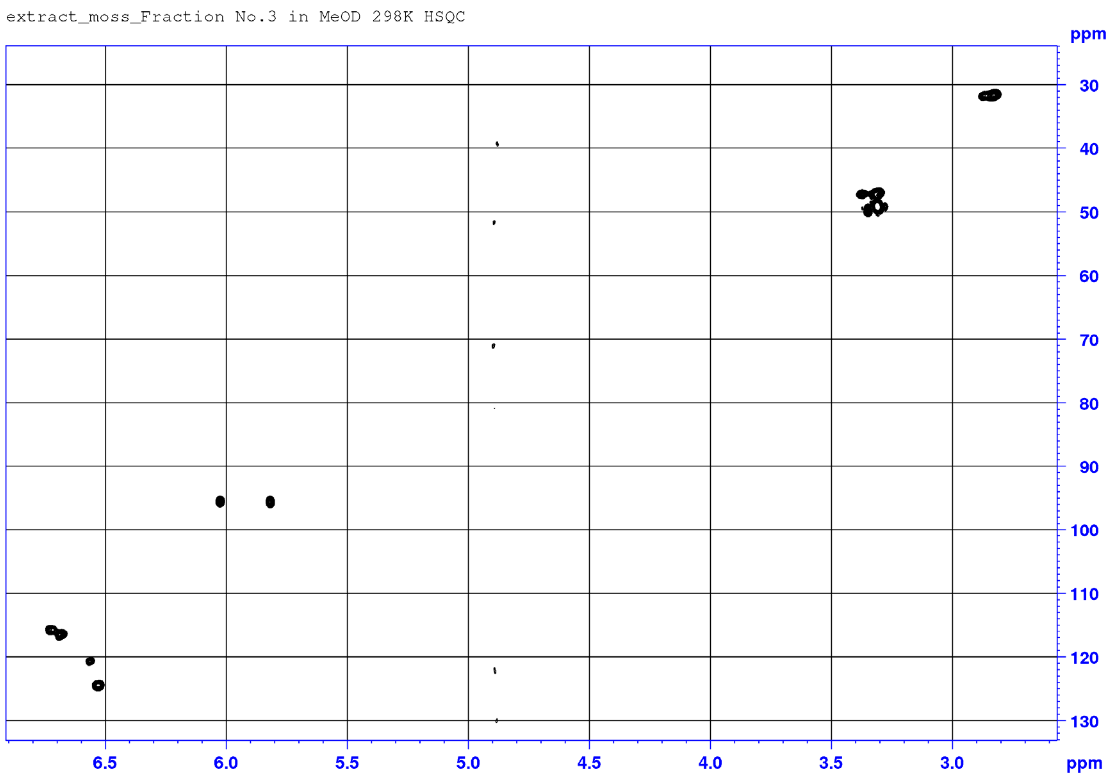

Figure S12. 2D ^{1}H-^{13}C HSQC NMR spectrum of fraction F3.

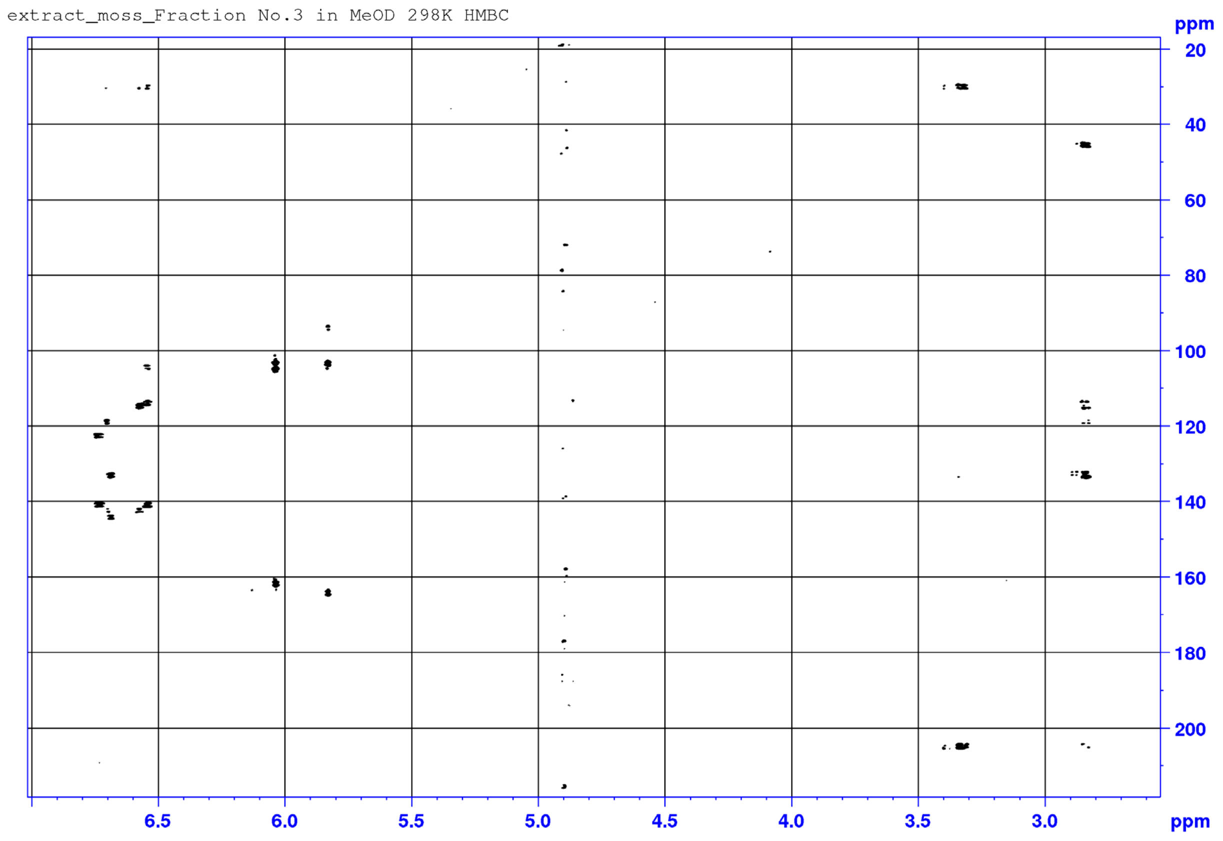

Figure S13. 2D ^{1}H-^{13}C HMBC NMR spectrum of fraction F3.

Protocol of QSAR/QSTR testing the 3-hydroxyphloretin trimer (predicted with the ACD/Labs Percepta software v. 2021.1.3, Advanced Chemistry Development, Toronto, ON, Canada).

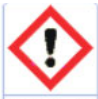

Cat. 4 | Cat. 5

78% probability that compound belongs to any of above categories

82% probability that LD50 =< 5000 mg/kg
95% probability that LD50 > 300 mg/kg
Probabilities of LD50 (mg/kg) ranges:

=< 5	=< 50	=< 300	=< 2000	=< 5000
0.002	0.011	0.047	0.429	0.822

> 5	> 50	> 300	> 2000	> 5000
0.998	0.989	0.953	0.571	0.178

- **Lethal Dose (LD50, mg/kg)**

Species/Administration route		LD50	Reliability
Mouse/Intraperitoneal<br>Library used in calculation:<br>*LD50 Mouse Intraperitoneal v. 1.2 (Read-only)*	Configure Train	500 mg/kg	Borderline (RI = 0.47)
Mouse/Oral<br>Library used in calculation:<br>*LD50 Mouse Oral v. 1.2 (Read-only)*	Configure Train	4900 mg/kg	Not Reliable (RI = 0.24)
Mouse/Intravenous<br>Library used in calculation:<br>*LD50 Mouse Intravenous v. 1.2 (Read-only)*	Configure Train	140 mg/kg	Borderline (RI = 0.39)
Mouse/Subcutaneous<br>Library used in calculation:<br>*LD50 Mouse Subcutaneous v. 1.2 (Read-only)*	Configure Train	0.37 mg/kg	Not Reliable (RI = 0.19)
Rat/Intraperitoneal<br>Library used in calculation:<br>*LD50 Rat Intraperitoneal v. 1.2 (Read-only)*	Configure Train	9900 mg/kg	Borderline (RI = 0.42)
Rat/Oral<br>Library used in calculation:<br>*LD50 Rat Oral v. 1.2 (Read-only)*	Configure Train	3100 mg/kg	Borderline (RI = 0.39)

- **Mutagenicity (Ames Test)**

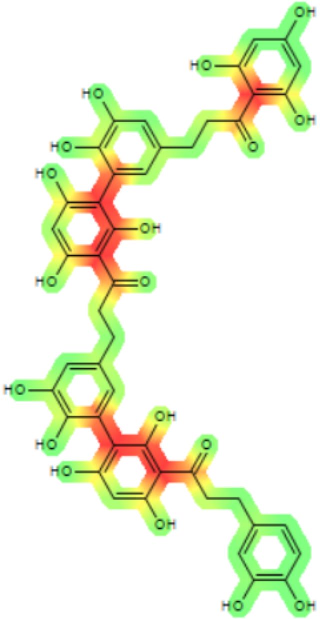

Probability of positive Ames test: 0.13
Reliability: Borderline (RI = 0.36)

Library used in calculation:
AMES Test v. 1.3 (Read-only)

- **Probabilities of Health Effect**

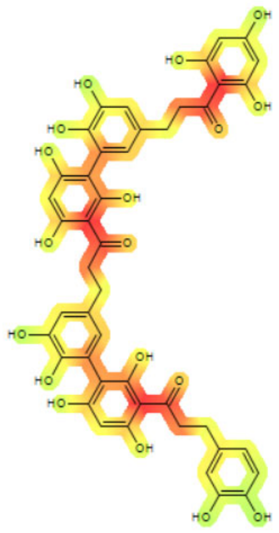

Blood	0.17
Cardiovascular system	0.84
Gastrointestinal system	0.90
Kidney	0.67
Liver	0.65
Lungs	0.89

- **Eye Irritation (Draize Test): 0.03**
- **Skin Irritation (Draize Test): 0.12**

**PhysChem Profiling**		
LogP	5.56	Very lipophilic
MW	866.77	Bad
H-Donors	15	Bad
H-Acceptors	18	Bad
Rot. Bonds	14	Bad
Rings	6	Bad
Lipinski	4 violations	Bad
Lead-like	4 violations	Bad
Solubility	0.001 mg/ml	Highly insoluble
**ADME Profiling**		
Caco-2	Pe = 0.1E-6 cm/s	Poorly permeable
PPB	100%	Extensively bound
CNS	Score = -9.39	Non-penetrant
HIA	34%	Moderately absorbed
Metabolic Stability	0.41	Undefined
**Drug Safety Profiling**		
P-gp Substrates	0.36	Undefined
CYP1A2 Inhibitor	0.66	Undefined
CYP2C9 Inhibitor	0.73	Efficient inhibitor
CYP2C19 Inhibitor	0.72	Inhibitor
CYP2D6 Inhibitor	0.43	Undefined
CYP3A4 Inhibitor	0.73	Inhibitor
Ames	0.37	Undefined
hERG	0.19	Non-inhibitor
